# Pure curcumin decreases the expression of WT1 by upregulation of miR-15a and miR-16-1 in leukemic cells

**DOI:** 10.1186/1756-9966-31-27

**Published:** 2012-03-27

**Authors:** Shen-meng Gao, Jun-jun Yang, Chi-qi Chen, Jun-jie Chen, Li-ping Ye, Lu-yao Wang, Jian-bo Wu, Chong-yun Xing, Kang Yu

**Affiliations:** 1 Laboratory of Internal Medicine, The First Affiliated Hospital of Wenzhou Medical College, 2 FuXue Road, Wenzhou 325000, China; 2 Clinical Laboratory, The Second Affiliated Hospital of Wenzhou Medical College, 109 Xuanyuanxi Road, Wenzhou 325000, China; 3 Department of Hematology, The First Affiliated Hospital of Wenzhou Medical College, 2 FuXue Road, Wenzhou 325000, China

**Keywords:** Curcumin, WT1, miR-15a, miR-16-1

## Abstract

**Background:**

Pure curcumin has been reported to down-regulate the expression of WT1 in leukemic cells. However, the molecular mechanism underlying the down-regulation of WT1 by curcumin is not completely delineated. The purpose of this present study is to identify a new miRNA-mediated mechanism which plays an important role in the anti-proliferation effects of curcumin in leukemic cells.

**Methods:**

K562 and HL-60 cells were treated with different concentrations of curcumin for 24 and 48 hours, the level of miR-15a/16-1 and WT1 were detected by qRT-PCR and Western blotting. WT1 expression and cell proliferation were detected by Western blotting and CCK-8, after curcumin treated-K562 and HL-60 cells were transfected with anti-miR-15a/16-1 oligonucleotides.

**Results:**

We found that pure curcumin upregulated the expression of miR-15a/16-1 and downregulated the expression of WT1 in leukemic cells and primary acute myeloid leukemia (AML) cells. Overexpression of miR-15a/16-1 deduced the protein level of WT1 in leukemic cells, but downregulation of WT1 by siRNA-WT1 could not increase the expression of miR-15a/16-1 in leukemic cells. These results reveal that curcumin induced-upregulation of miR-15a/16-1 is an early event upstream to downregulation of WT1. Furthermore, anti-miR-15a/16-1 oligonucleotides (AMO) partly reversed the downregulation of WT1 induced by pure curcumin in leukemic cells and AMO promoted the growth of curcumin treated-K562 and HL-60 cells.

**Conclusion:**

Thus, these data suggest for the first time that pure curcumin downregulated the expression of WT1 partly by upregulating the expression of miR-15a/16-1 in leukemic cells. miR-15a/16-1 mediated WT1 downregulation plays an important role in the anti-proliferation effect of curcumin in leukemic cells.

## Introduction

The Wilms' tumor 1 (WT1) gene, which is located at the short arm of chromosome 11 and contains 10 exons, encodes a DNA-binding transcription factor essential for embryonal development [1]. High level of WT1, which is detected in most cases of acute human leukemia and chronic myelogeous leukemia (CML) in blast crisis, is associated with a worse long-time prognosis [2]. Downregulation of WT1 by special siRNA can inhibit cell proliferation and induce apoptosis in K562 and HL-60 cells [3]. WT1 acts as a potent transcriptional regulation factor involved in cell growth and development due to the presence of zinc fingers [4]. WT1 is firstly thought to function as tumor suppressor, but the following wildly studies support that WT1 acts as oncogene [5].

Curcumin, a naturally occurring flavinoid and proapoptotic compound derived from the rhizome of *Curcuma longa*, has strong anti-inflammatory, antioxidant, anticarcinogen, anticancer properties through regulating multiple downstream cancer-related signaling molecules. The molecular targets of curcumin include modulation of NF-kappaB, Jak/STAT, WT1, extracellular signal regulated kinase and other key molecules involved in tumorigenesis [6-8]. The mechanisms underlying the anticancer activity of curcumin have been widely investigated. Bharti *et al. *showed curcumin decreased NF-kappaB in human multiple myeloid cells, leading to the suppression of proliferation and induction of apoptosis [7]. Recently more and more data have shown that WT1 is a very important target gene by curcumin [9]. However the exact mechanism by which curcumin downregulated the expression of WT1 is still not clear.

MicroRNAs (miRNAs) are non-coding regulatory RNAs of 21 to 25 nucleotides which regulate most of basal progress such as cell proliferation, survival, apoptosis, and differentiation by triggering either translational repression or mRNA degradation [10]. Furthermore, computational prediction demonstrated that each miRNA may target hundreds of genes, and that more than 50% of human protein-coding genes could be modulated by miRNAs [11]. Recently some data have indicated pure curcumin inhibited cancer cell proliferation though miRNAs mediated signal pathway. Michael *et al. *showed curcumin inhibited the proliferation of pancreatic cancer cells through upregulation of miR-22 and downregulation of miR-199a* [12]. Yang *et al. *demonstrated that curcumin induced MCF-7 cells apoptosis through miR-15a/16-1 mediated down-regulation of Bcl-2 [13]. These emerging results suggest that specific targeting of miRNAs by natural agents may open new avenues for the complete elucidation of antitumor activity by curcumin.

In this study, we explored the potential modulation of miR-15a and miR-16-1 by curcumin in leukemic cells. Our study aims to explain a new mechanism by which curcumin downregulates the expression of WT1 via the upregulation of miR-15a/16-1 in leukemic cells.

## Material and methods

### Cell lines and primary AML cells

Leukemic cell lines (K562 and HL-60) were employed for the present study. All cells were cultured in RPMI 1640 supplemented with 10% heat-inactivated fetal bovine serum (Invitrogen, CA, USA) in humidified 37°C incubator with 5% CO2. Primary leukemic cells were obtained from 12 patients with acute myeloid leukemia (AML) (3 M2, 2 M3, 3 M4 and 4 M5, The First Affiliated Hospital of Wenzhou Medical College) with informed consent. The detailed data of the patients were showed in Table 1. The diagnosis was established according to French-American-British classification. All manipulations were approved by the Medical Science Ethic Committee of Wenzhou Medical College. All these patients did not receive any chemical therapy treatments. Primary leukemic cells were isolated by Ficoll density gradient centrifugation (GE Healthcare, Uppsala, Sweden). Pure curcumin (Sigma-Aldrich, St Louis, MO) was dissolved in DMSO as 20 mM stock solution and kept at -20°C. For experiments, leukemic cells and primary AML cells were cultured in serial concentrations of curcumin and control cultures were treated with DMSO only.

**Table 1:** The data of acute myeloid leukemia patients

NO	Sex	Age(y)	FAB subtype	Chromosome karyotype
1	M	24	M5	46, XY
2	M	36	M3	46, XY PML-RARa+
3	F	47	M5	46, XX
4	F	53	M4	46, XX MYH11-CBFβ+
5	M	29	M3	46, XY PML-RARa+
6	F	48	M2	46, XX AML-ETO+
7	F	35	M4	46, XX MYH11-CBFβ+
8	M	41	M5	46, XY
9	F	58	M2	46, XX AML-ETO+
10	M	47	M4	46, XY
11	M	41	M2	46, XY
12	F	26	M5	46, XX

### Plasmids transfection

pRETROSUPER vector expressing miR-15a/16-1 (pRS-15/16) was constructed as previously described. The same empty plasmid (pRS-E) was served as negative control. K562 and HL-60 cells were transiently transfected with 1 μg/mL (final concentration) pRS-15/16 or pRS-E vector mediated by Lipofectamine™ LTX and PLUS™ Reagents (Invitrogen) according to the manufacturer's instructions.

### RNA extraction

Total RNA from curcumin-treated or untreated leukemic cells were extracted by TRIzol (Invitrogen) Following the manufacture's protocol. RNA concentration and quality were quantified by measuring the absorbance at 260 nm with Beckman DU6400 spectrophotometer (Beckman, USA) and gel analysis.

### qPCR for miRNA and mRNA expression

Quantitative real-time polymerase chain reaction(qRT-PCR) analysis for miR-15a and miR-16-1 was performed in triplicate by the aid of the NCode™ miRNA First-strand cDNA synthesis (Invitrogen) and SYBR^® ^Green PCR Master Mix (Applied Biosystems, Foster City, CA) according to the manufacturer's instructions. U6 snRNA level was used for normalization. The fold change for each miRNA in curcumin-treated leukemic cells relative to untreated cells was calculated using the 2^-ΔΔCT ^method [14]. WT1 transcript was determined by quantitative real-time PCR using specific primer. ABL and GAPDH housekeeping genes were used for normalization [15,16]. The following primers were used respectively, miR-15a: 5'-TAG CAG CAC ATA ATG GTT TGT G-3', miR-16-1: 5'-TAG CAG CAC GTA AAT ATT GGC G-3', U6: 5'-CGC AAG GAT GAC ACG CAA ATT C-3', WT1: sense strand: 5'-CAG GCT GCA ATA AGA GAT ATT TTA AG CT-3', antisense strand: 5'-GAA GTC ACA CTG GTA TGG TTT CTC A-3', Taqman probe: 5'-Fam-CTT ACA GAT GCA CAG CAG GAA GCA CAC TGA-Tamra-3'), ABL: (sense strand: 5'-GAT GTA GTT GCT TGG GAC CCA-3', antisense strand: 5'-TGG AGA TAA CAC TCT AAG CAT AAC TAA AGG T-3', Taqman probe: 5'-Fam-CCA TTT TTG GTT TGG GCT TCA CAC CAT T-Tamra-3'). GAPDH: (sense strand: 5'-CCA GGT GGT CTC CTC TGA CTT C-3', antisense strand: 5'-GTG GTC GTT GAG GGC AAT G-3', Taqman probe: 5'- Fam-ACA GCG ACA CCC ACT CCT CCA CCT T-Tamra-3').

### Cell counting kit-8 (CCK-8) assay

K562 and HL-60 cells were seeded into 96-well plates (6.0 × 10^3 ^cells/well). Cell viability was assessed by CCK-8 assay (Dojin Laboratories, Kumamoto, Japan). The absorbance at 450 nm (A450) of each well was read on a spectrophotometer. Three independent experiments were performed in quadruplicate.

### Western blotting

Protein extracts from cell lines, patient samples prepared with RIPA lysis buffer (50 mM TrisHCl, 150 mM NaCl, 0.1% SDS, 1% NP-40, 0.5% sodiumdeoxycholate, 1 mM PMSF, 100 mM leupeptin, and 2 mg/mL aprotinin, pH 8.0) were separated on an 8% SDS-polyacrylamide gel and transferred to nitrocellulose membranes. After blocking with 5% nonfat milk, the membranes were incubated with an appropriate dilution (WT1 1:2000) of the primary antibody (Abcom, Cambridge, MA, USA), followed by incubation with the horseradish peroxidase (HRP)-conjugated secondary antibody (Abcom). The signals were detected by chemiluminescence phototope-HRP kit (Cell Signaling, Danvers, MA, USA). Blots were stripped and reprobed with anti-GAPDH antibody (Abcom) as an internal control. All experiments were repeated three times.

### siRNA, mimics, and anti-miR-15a/16-1 oligonucleotide (AMO) transfection

SiRNA sequences targeting WT1: ccauaccagugugacuuca corresponds to positions 9-27 of exon 7 within the WT1 coding sequence. SiRNA-WT1 and unspecific control siRNA (N.C) were synthesized from Invitrogen. 50 nM SiRNA-WT1 or N.C were transfected into K562 and HL-60 cells using Hiperfect transfection reagent (Qiagen, Valencia, USA) according to manufacturer's instructions. miR-15a or miR-16-1 mimics was synthesized from Gene Pharma (Shanghai, China). 40 uM miR-15a or miR-16-1 mimics were transfected into K562 using Hiperfect transfection reagent (Qiagen). The sequences of AMO were designed according to the principle of sequences complementary to mature miRNA-15a/16-1. AMO and scramble (SCR) were chemically synthesized by Qiagen. AMO and SCR (final concentration of 50 nM) were transfected into K562 and HL-60 cells using the Hiperfect transfection reagent (Qiagen). All transfections were performed in triplicate for each time point.

### Statistical analysis

The significance of the difference between groups was determined by Student's *t*-test. A *P *value of less than .05 was considered statistically significant. All Statistical analyses were performed with SPSS software (version 13).

## Results

### Pure curcumin downregulated the expression of WT1 and effectively inhibited cell proliferation in leukemic cells

**Figure 1:**
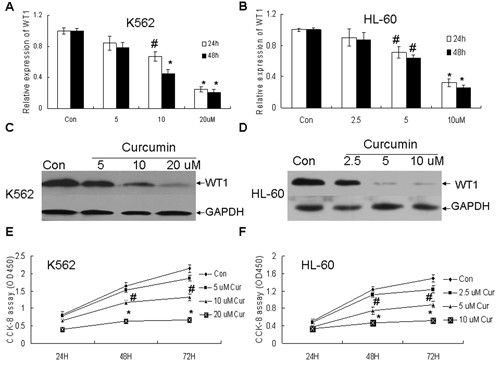
**Pure curcumin down-regulated the expression of WT1 and inhibited the proliferation in K562 and HL-60 cells**. (A and C) K562 cell was treated with non-cytotoxic doses of pure curcumin (5, 10, 20 uM) for 24 and 48 hours, then the mRNA level of WT1 was detected by qRT-PCR and the protein level of WT1 was detected by Western blotting after curcumin treatment for 48 hours. (B and D) HL-60 cell was treated with non-cytotoxic doses of pure curcumin (2.5, 5, 10 uM) for 24 and 48 hours, then the mRNA level of WT1 was detected by qRT-PCR and the protein level of WT1 was detected by Western blotting. GAPDH as loading control. (E and F) CCK8 assay was performed when K562 and HL-60 cells were treated for indicated concentration of curcumin for 24, 48 and 72 hours. ^# ^and *represent less than 0.05 and 0.01 of *P*-values, respectively, as compared to control.

As reported previously [17], low concentration of pure curcumin could inhibit the growth of leukemic cells and downregulate the expression of WT1. The mRNA and protein levels of WT1 were detected by qRT-PCR and Western blotting respectively after K562 and HL-60 cells were treated with non-cytotoxic doses of pure curcumin (5, 10, 20 uM for K562 and 2.5, 5, 10 uM for HL-60) [17]. As indicated in Figure 1A-D pure curcumin downregulated the expression of WT1 in time- and concentration -dependent manner. The mRNA levels of WT1 in the K562 cells were decreased by 12%, 55%, and 73% in response to treatment with 5, 10, and 20 μM curcumin at 48 hours compared with the vehicle control (Figure 1A). To test whether ABL housekeeping gene was regulated by curcumin, another widely used housekeeping gene GAPDH was used for normalization. As Additional file 1: Figure S1A demonstrated no difference occurred in WT1 expression between GAPDH and ABL for normalization. Meanwhile the protein levels of WT1 in the k562 cells were significantly decreased after 10 and 20 uM curcumin treatment at 48 hours (Figure 1C). In HL-60 cells 5 and 10 uM curcumin also significantly downregulated the mRNA and protein levels of WT1 (Figure 1B and 1D). Finally CCK-8 assay showed that low concentrations of pure curcumin could effectively inhibit the growth of leukemic cells (Figure 1E and 1F).

### Pure curcumin upregulated the expression of miR-15a/16-1 in leukemic cells and primary AML blasts

**Figure 2:**
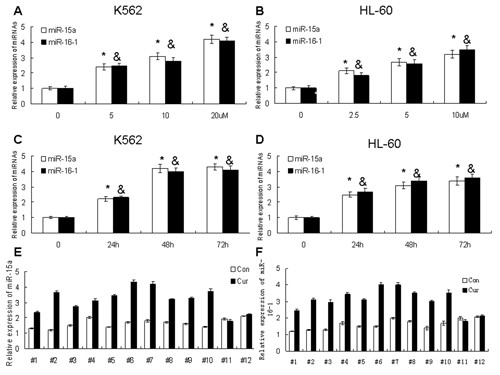
**Pure curcumin upregulated the expression of miR-15a/16-1 in leukemic cell lines and primary AML blasts**. (A and C) The expression of miR-15a and miR-16-1 were detected by qRT-PCR after K562 and HL-60 cells were treated with different concentration of curcumin for 48 hours. (B and D) K562 and HL-60 cells were treated with 20 uM or 10 uM curcumin respectively for 24, 48, and 72 hours, then the relative expressions of miR-15a and miR-16-1 were detected by qRT-PCR. Data are shown as mean ± SD from three independent experiments. (E and F) Primary leukemic cells were isolated by Ficoll density gradient centrifugation and were treated with 20 uM pure curcumin for 48 hours, then the levels of miR-15a and miR-16-1 were detected by qRT-PCR. ^# ^and ^&^represent less than 0.01 of *P*-values as compared to control.

Although pure curcumin decreased the expression of WT1 in K562 and HL-60 cells, the exact mechanism is still unkown. miRNAs are very important for gene expression. Calin *et al. *reported that miR-15a/16-1 downregulate the protein level of WT1 in MEG-01 cells [18]. Taking these into consideration we want to explore whether pure curcumin can regulate the expression of miR-15a/16-1 in leukemic cells. The levels of miR-15a and miR-16-1 were detected by qRT-PCR after K562 and HL-60 cells were treated with indicated doses of pure curcumin. As indicated in Figure 2A-D pure curcumin could upregulate the expression of miR-15a/16-1 almost 2-3 folds than untreated groups in time- and concentration-dependent manner in K562 and HL-60 cells. To test whether the upregulation of miR-15a/16-1 induced by curcumin also occurred in primary leukemic cells, Primary leukemic cells of 12 AML patients were separated by Ficoll and were treated with 20 uM pure curcumin for 48 hours. The upregulation of miR-15a/16-1 was observed in 10 of 12 patients (Figure 2E and 2F). This data indicate that pure curcumin can upregulate the expression of miR-15a and miR-16-1 in leukemic cell lines and primary AML cells. To prove whether WT1 is downregulated by curcumin in primary AML cells, WT1 expression is detected by real time RT-PCR in the same 12 patients. As Additional file 1: Figure S1B demonstrated the downregulation of WT1 was observed in 8 of 12 patients. In patients 5 and 10, curcumin upregulated the expression of miR-15a and miR-16-1 but did not downregulate the expression of WT1.

### Overexpression of miR-15a/16-1 could deduce WT1 expression but downregulation of WT1 by siRNA could not increase the expression of miR-15a/16-1 in leukemic cells

**Figure 3:**
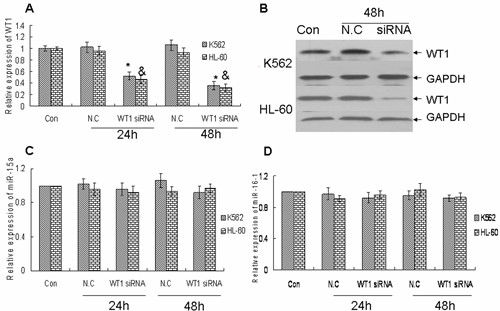
**Down-regulation of WT1 by siRNA could not increase the expression of miR-15a/16-1 in leukemic cells**. (A and B) K562 and HL-60 cells were transfected with 50 nM siRNA-WT1, 50 nM N.C or neither of the above for 24 and 48 hours, then the relative mRNA expression of WT1 and the corresponding WT1 protein were respectively measured by quantitative real-time PCR and Western blotting. GAPDH as loading control. (C and D) The relative expressions of miR-15a and miR-16-1 were measured by qRT-PCR after K562 and HL-60 cells were transfected with 50 nM siRNA-WT1, 50 nM N.C or neither of the above for 24 and 48 hours. * and ^&^*P *< 0.01 versus negative control (N.C).

Our previous data showed overexpression of miR-15a/16-1 obviously reduced the protein level of WT1 after transfection with pRS-15/16 compared with normal controls in K562 and HL-60 cells, whereas the level of WT1 mRNA was not significantly affected [19]. To prove whether single miR-15a or miR-16-1 could downregulated the expression of WT1, WT1 protein level was detected by Western blotting after miR-15a or miR-16-1 mimics were transfected into K562 cells. As demonstrated in Additional file 1: Figure S1C, both miR-15a and miR-16-1 could downregulated the expression of WT1. Although curcumin could upregulate the expression of miR-15a/16-1 and downregulate the expression of WT1, whether the upregulation of miR-15a/16-1 was caused by the downregulation of WT1 is unknown. The siRNA specific for WT1 was used to mimick the downregulation of WT1 by curcumin. WT1 mRNA and protein levels were estimated by quantitative real-time PCR and Western blotting individually after K562 and HL-60 cells were transfected with siRNA-WT1 or negative control for 24 and 48 hours. WT1 siRNA-treated K562 and HL-60 cells showed a significant reduction of WT1 mRNA level as compared to control cells (Figure 3A). Furthermore the reduction of mRNA using siRNA resulted in a markedly decrease of WT1 protein level after 48 hours in K562 and HL-60 cells (Figure 3B). Finally we observed that the level of miR-15a and miR-16-1 were not significantly altered by siRNA-WT1 compared with normal control (Figure 3C and 3D). All these data demonstrate that downregulation of WT1 can not affect the expression of miR-15a and miR-16-1 in K562 and HL-60 cell lines. These data strongly indicate that pure curcumin induced-upregulation of miR-15a/16-1 is an event upstream to the downregulation of WT1.

### Anti-miR-15a/16-1 oligonucleotides (AMO) partly reversed the down-regulation of WT1 induced by curcumin in leukemic cells

**Figure 4:**
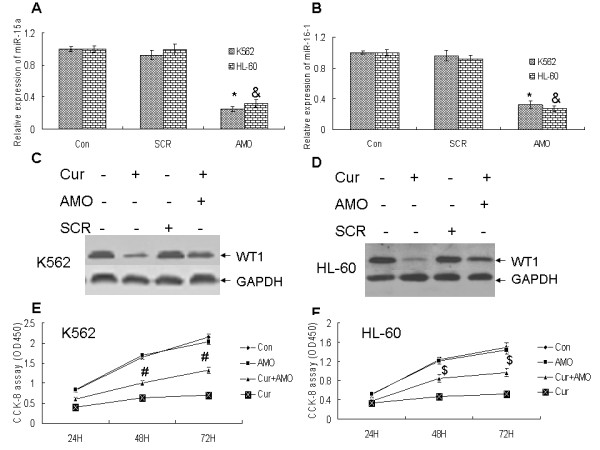
**Anti-miR-15a/16-1 oligonucleotides (AMO) partly reversed the downregulation of WT1 induced by curcumin in K562 and HL-60 cells**. (A and B) The relative expressions of miR-15a/16-1 were measured by qRT-PCR after K562 and HL-60 cells were transfected with 50 nM of anti-miR-15a/16-1 oligonucleotides for 48 hours. * and ^&^*P *< 0.01 versus negative control (SCR). (C and D) 20 uM curcumin treated-K562 and 10 uM curcumin treated- HL-60 cells were transfected with 50 nM of anti-miR-15a/16-1 oligonucleotides for 48 hours, then the protein levels of WT1 were measured by Western blotting. GAPDH as loading control. (E and F) 20 uM curcumin treated-K562 and 10 uM curcumin treated- HL-60 cells were transfected with 50 nM of anti-miR-15a/16-1 oligonucleotides for 24, 48, and 72 hours, then cell proliferation was measured by CCK-8 assay. ^# ^and ^$ ^represent less than 0.05 of p-values, compared respectively with pure curcumin treatment alone at the same time.

To further confirm that pure curcumin down-regulated the expression of WT1 by up-regulation of miR-15a/16-1, 20 uM curcumin treated-K562 and 10 uM curcumin treated- HL-60 cells were transfected with 50 nM anti-miR-15a/16-1 oligonucleotides for 48 hours. The levels of WT1 protein were detected by Western blotting after transfection. As Figure 4A and 4B demonstrated that anti-miR-15a/16-1 oligonucleotides could effectively decrease the expression of miR-15a and miR-16-1 in K562 and HL-60 cells. Moreover, anti-miR-15a/16-1 oligonucleotides partly abolished the inhibitory effect of curcumin on WT1 protein expression (Figure 4C and 4D). Finally, as indicated in Figure 4E and 4F, 20 uM curcumin treated-K562 and 10 uM curcumin treated-HL-60 cells were transfected with 50 nM of anti-miR-15a/16-1 oligonucleotides for 24, 48 and 72 hours, the CCK-8 assay revealed that anti-miR-15a/16-1 oligonucleotides effectively reversed the inhibition of cell proliferation caused by curcumin in K562 and HL-60 cells.

## Discussion

WT1 is considered to play an important role in leukemogenesis because the expression of WT1 increases 1000-10000 fold in primary leukemic cells than normal cells [20]. Glienke and Bergmann showed that siRNA-reduced WT1 mRNA expression was associated with a decreased cell proliferation in K562 and HL-60 cells after transfection for 24 and 48 h [3]. Several studies indicated that pure curcumin downregulated the expression of WT1 in leukemic cell lines [9]. Moreover, combined treatment with curcumin and siRNA targeting WT1 resulted in a significant inhibition of cell proliferation compared to curcumin-treated cells alone in pancreatic cancer cells. All these data suggest that WT1 plays an important role in the anti-proliferative effects of curcumin. However, the mechanism by which pure curcumin downregulates WT1 expression is still unknown. Our data show for the first time that pure curcumin downregulates WT1 expression via miRNAs pathway.

The gene expression is regulated via a complicated network. Semsri *et al. *reported that pure curcumin decreased the mRNA and protein levels of WT1 through attenuating WT1 auto-regulatory function and inhibiting PKCalpha signaling in K562 cells [21]. Our data showed that curcumin downregulated the expression of WT1 via miRNAs mediated pathway. However, whether other regulating factors are involved in the regulation is still not completely delineated. Therefore it is difficult to accurately calculated how much of the down-regulation of WT1 in the curcumin- treated cells is attributable to the action of the miRNAs. Our previous data had showed overexpression of miR-15a/16-1 downregulated the protein level of WT1 but not mRNA level [19]. However, in this report curcumin decreased the mRNA and protein levels of WT1 in leukemic cells. Therefore, it is obvious that additional mechanisms [21] other than the induction of miR-15a/16-1 expression contribute to curcumin-induced WT1 downregulation. Taken together, as Additional file 1: Figure S2 indicated pure curcumin inhibited the cell growth partly through miR-15a/16-1 mediated downregulation of WT1.

Each miRNA typically targets mRNAs of hundreds of distinct genes by pairing to the mRNAs of protein-coding genes. Previous data had reported that Bcl-2 [18], WT1 [18], caprin-1 [22] and HMGA1 [22] were the target genes by miR-15a/16-1. WT1 and Bcl-2 are highly expressed in leukemic cells and function as oncogenes. The use of SiRNAs against WT1 and Bcl-2 in leukemic cells could effectively inhibit leukemic cells growth [3]. Overexpression of miR-15a/16-1 in leukemic cells suppressed cell growth probably through targeting WT1 and Bcl-2. However it is difficult to estimate how much of the inhibition of cell growth in leukemic cells is attributable to the downregulation of WT1 or Bcl-2.

Recent studies have shown that natural agents, including curcumin, isoflavone, and EGCG, can regulate the expression of many miRNAs which increase the sensitivity of cancer cells to conventional agents and thereby suppress tumor cell proliferation [23,24]. Zhang *et al. *reported that pure curcumin downregulated the expression of miR-186* in A549/DDP cells. Moreover, overexpression of miR-186* significantly inhibited curcumin-induced apoptosis in A549/DDP cells and transfection of cells with a miR-186* inhibitor promoted A549/DDP apoptosis [25]. Mudduluru *et al. *demonstrated that in Rko and HCT116 cells curcumin reduced the expression of miR-21 in a dose-dependent manner by inhibiting AP-1 binding to the promoter of miR-21, and induced the expression of the tumour suppressor programmed cell death protein 4, which is a target of miR-21 [26]. These data showed curcumin suppress tumor cell growth through downregulating a panel of onco-miRNAs. Saini *et al. *showed curcumin increased the expression of miR-203 via inducing the hypomethylation of the miR-203 promotes. This led to downregulation of miR-203 target genes Akt2 and Src resulting in decreased proliferation and increased apoptosis in bladder cancer cells [27]. Bao *et al. *demonstrated that a novel curcumin analog CDF inhibited pancreatic tumor growth and aggressiveness through upregulating a panel of tumor suppressive miRNAs let-7, miR-26a, miR-101 and attenuating EZH2 expression [28]. In a word curcumin suppress tumor cell growth through downregulating a panel of onco-miRNAs or upregulating a panel of tumor suppressive miRNAs. However, very little data reported that miRNAs besides miR-15a/16-1 could regulate the expression of WT1. More study were required to prove whether other miRNAs which target WT1 were regulated by curcumin.

Recently it has been reported that curcumin is an epigenetic agent. Curcumin inhibits the activity of DNA methyltransferase I (DNMT1) through covalently blocking the catalytic thiolate of C1226 of DNMT1. Global DNA methylation levels were decreased by approximately 20% in a leukemic cell line which is treated with 30 uM curcumin compared with untreated basal methylation levels [29]. Curcumin can also modulates histone acetyltransferases (HAT) and histone deacetylases (HDACs) [30]. Previous data had indicated that curcumin upregulated the levels of miR-15a and miR-16-1 in MCF-7 and other cells [13]. Since curcumin is a DNA hypomethylation agent, epigenetic modulation of microRNA expression may be an important mechanism underlying biological effects of curcumin. Curcumin probably regulates the expression of miR-15a/16-1 through epigenetic modulation.

Overexpression of miR-15a and 16-1 downregulated the expression of WT1. Calin *et al. *showed that WT1 was a target gene of miR-15a/16-1 in MEG-01 cells by microarray and proteomics analysis [18]. However, whether WT1 was directly targeted by miR-15a and miR-16-1 in leukemic cells was not verified in lab. Our previous data showed that overexpression of miR-15a and miR-16-1 in K562 and HL-60 cells significantly downregulated the protein level of WT1. However the mechanism of miR-15a/16-1 downregulating WT1 protein level is not through targeting mRNAs according to the degree of complementarity with their 3'untranlation region. In conclusion, miR-15a and miR-16-1 probably regulated WT1 expression through an indirect effect on WT1 [19].

Anti-miR-15a/16-1 has the ability to efficiently and specifically silence endogenous miR-15a and miR-16-1. Our data showed anti-miR-15a/16-1 could partly reverse the expression of WT1 in curcumin-treated K562 and HL-60 cells. These results suggest that the decrease of WT1 expression is partly attributable to the increased expression of miR-15a and miR-16-1 in curcumin-treated leukemic cells. Thus our data suggest that one of the important anti-proliferation effects of curcumin on leukemic cells is via miRNAs pathway. Given that many miRNAs are regulated by pure curcumin, many further experiments will be required to define other miRNAs besides miR-15a/16-1 are regulated by curcumin and play an important role in anti-tumor effects of curcumin.

## Conclusion

Therefore, we conclude that pure curcumin can decrease WT1 expression partly through upregulating the expression of miR-15a and miR-16-1. Our data show for the first time that miRNAs pathway plays an important role in the function of anti-proliferation by pure curcumin in leukemic cells.

## Conflict of interests

The authors declare that they have no competing interests.

## Authors' contributions

SMG and JJY contributed to samples collection, cell culture and drafted manuscript. CQC and JJC carried out Western blotting. LPY and LYW carried out plasmids, siRNA, and AMO transfection. JBW carried out CCK8 and qRT-PCR. CYX carried out clinical data collection. KY performed the study design, statistical analysis, and manuscript writing. All authors read and approved the final manuscript.

## Supplementary Material

Additional file 1 **Figure S1**. (A) K562 cells were treated with 5, 10, 20 uM pure curcumin for 48 hours, then the mRNA level of WT1 was detected by qRT-PCR. ABL and GAPDH served as different housekeeping for normalization. (B) Primary leukemic cells of 12 AML patients were separated by Ficoll and were treated with 20 uM pure curcumin for 48 hours, then the mRNA levels of WT1 were detected by qRT-PCR. (C) The protein level of WT1 was detected by Western blotting after negative control(N.C), miR-15a and miR-16-1 mimics were transfected into K562 for 48 hours. Figure S2. An illustration of the potential mechanisms of curcumin action in leukemic cells. Curcumin upregulated the expression of miR-15a/16-1 in leukemic cells. Overexpression of miR-15a/16-1 obviously reduced the protein level of WT1. However, downregulation of WT1 by siRNA could not increase the expression of miR-15a/16-1. These events showed that curcumin induced-upregulation of miR-15a/16-1 was an event upstream to the downregulation of WT1. Finally anti-miR-15a/16-1 oligonucleotides (AMO) partly reversed the down-regulation of WT1 induced by curcumin in leukemic cells and reversed the inhibition of cell proliferation caused by curcumin in K562 and HL-60 cells. Click here for file

## References

[B1] Kreidberg JA Sariola H Loring JM Maeda M Pelletier J Housman D Jaenisch R WT-1 is required for early kidney development Cell 1993 74 679 691 10.1016/0092-8674(93)90515-R 8395349

[B2] Bergmann L Miething C Maurer U Brieger J Karakas T Weidmann E Hoelzer D High levels of Wilms' tumor gene (wt1) mRNA in acute myeloid leukemias are associated with a worse long-term outcome Blood 1997 90 1217 1225 9242555

[B3] Glienke W Maute L Koehl U Esser R Milz E Bergmann L Effective treatment of leukemic cell lines with wt1 siRNA Leukemia 2007 21 2164 2170 10.1038/sj.leu.2404878 17690705

[B4] Dame C Kirschner KM Bartz KV Wallach T Hussels CS Scholz H Wilms tumor suppressor, Wt1, is a transcriptional activator of the erythropoietin gene Blood 2006 107 4282 4290 10.1182/blood-2005-07-2889 16467207

[B5] Morrison AA Viney RL Ladomery MR The post-transcriptional roles of WT1, a multifunctional zinc-finger protein Biochim Biophys Acta 2008 1785 55 62 17980713 10.1016/j.bbcan.2007.10.002

[B6] Kuttan R Bhanumathy P Nirmala K George MC Potential anticancer activity of turmeric (Curcuma longa) Cancer Lett 1985 29 197 202 10.1016/0304-3835(85)90159-4 4075289

[B7] Bharti AC Donato N Singh S Aggarwal BB Curcumin (diferuloylmethane) down-regulates the constitutive activation of nuclear factor-kappa B and IkappaBalpha kinase in human multiple myeloma cells, leading to suppression of proliferation and induction of apoptosis Blood 2003 101 1053 1062 10.1182/blood-2002-05-1320 12393461

[B8] Glienke W Maute L Wicht J Bergmann L Wilms' tumour gene 1 (WT1) as a target in curcumin treatment of pancreatic cancer cells Eur J Cancer 2009 45 874 880 10.1016/j.ejca.2008.12.030 19196508

[B9] Anuchapreeda S Tima S Duangrat C Limtrakul P Effect of pure curcumin, demethoxycurcumin, and bisdemethoxycurcumin on WT1 gene expression in leukemic cell lines Cancer Chemother Pharmacol 2008 62 585 594 10.1007/s00280-007-0642-1 18034345

[B10] Bartel DP MicroRNAs: genomics, biogenesis, mechanism, and function Cell 2004 16 281 297 14744438 10.1016/s0092-8674(04)00045-5

[B11] Lim LP Microarray analysis shows that some microRNAs downregulate large numbers of target mRNAs Nature 2005 433 769 773 10.1038/nature03315 15685193

[B12] Sun M Estrov Z Ji Y Coombes KR Harris DH Kurzrock R Curcumin (diferuloylmethane) alters the expression profiles of microRNAs in human pancreatic cancer cells Mol Cancer Ther 2008 7 464 473 10.1158/1535-7163.MCT-07-2272 18347134

[B13] Yang J Cao Y Sun J Zhang Y Curcumin reduces the expression of Bcl-2 by upregulating miR-15a and miR-16 in MCF-7 cells Med Oncol 2010 27 1114 1118 10.1007/s12032-009-9344-3 19908170

[B14] Livak KJ Schmittgen TD Analysis of relative gene expression data using real-time quantitative PCR and the 2(-Delta Delta C(T)) Method Methods 2001 25 402 408 10.1006/meth.2001.1262 11846609

[B15] Cilloni D Gottardi E De Micheli D Serra A Volpe G Messa F Rege-Cambrin G Guerrasio A Divona M Lo Coco F Saglio G Quantitative assessment of WT1 expression by real time quantitative PCR may be a useful tool for monitoring minimal residual disease in acute leukemia patients Leukemia 2002 16 2115 2121 10.1038/sj.leu.2402675 12357365

[B16] Beillard E Pallisgaard N van der Velden VH Bi W Dee R van der Schoot E Delabesse E Macintyre E Gottardi E Saglio G Watzinger F Lion T van Dongen JJ Hokland P Gabert J Evaluation of candidate control genes for diagnosis and residual disease detection in leukemic patients using 'real-time' quantitative reverse-transcriptase polymerase chain reaction (RQ-PCR) - a Europe against cancer program Leukemia 2003 17 2474 2486 10.1038/sj.leu.2403136 14562124

[B17] Anuchapreeda S Thanarattanakorn P Sittipreechacharn S Chanarat P Limtrakul P Curcumin inhibits WT1 gene expression in human leukemic K562 cells Acta Pharmacol Sin 2006 27 360 366 10.1111/j.1745-7254.2006.00291.x 16490174

[B18] Calin GA Cimmino A Fabbri M Ferracin M Wojcik SE Shimizu M Taccioli C Zanesi N Garzon R Aqeilan RI Alder H Volinia S Rassenti L Liu X Liu CG Kipps TJ Negrini M Croce CM MiR-15a and miR-16-1 cluster functions in human leukemia Proc Natl Acad Sci USA 2008 105 5166 5171 10.1073/pnas.0800121105 18362358 PMC2278188

[B19] Gao SM Xing CY Chen CQ Lin SS Dong PH Yu FJ miR-15a and miR-16-1 inhibit the proliferation of leukemic cells by down-regulating WT1 protein level J Exp Clin Cancer Res 2011 30 110 10.1186/1756-9966-30-110 22133358 PMC3245444

[B20] Ostergaard M Olesen LH Hasle H Kjeldsen E Hokland P WT1 gene expression: an excellent tool for monitoring minimal residual disease in 70% of acute myeloid leukaemia patients - results from a single-centre study Br J Haematol 2004 125 590 600 10.1111/j.1365-2141.2004.04952.x 15147374

[B21] Semsri S Krig SR Kotelawala L Sweeney CA Anuchapreeda S Inhibitory mechanism of pure curcumin on Wilms' tumor 1 (WT1) gene expression through the PKCalpha signaling pathway in leukemic K562 cells FEBS Lett 2011 585 2235 2242 10.1016/j.febslet.2011.05.043 21658388

[B22] Kaddar T Rouault JP Chien WW Chebel A Gadoux M Salles G Ffrench M Magaud JP Two new miR-16 targets: caprin-1 and HMGA1, proteins implicated in cell proliferation Biol Cell 2009 101 511 524 10.1042/BC20080213 19250063

[B23] Davis CD Ross SA Evidence for dietary regulation of microRNA expression in cancer cells Nutr Rev 2008 66 477 482 10.1111/j.1753-4887.2008.00080.x 18667010

[B24] Li Y VandenBoom TG Kong D Wang Z Ali S Philip PA Sarkar FH Up-regulation of miR-200 and let-7 by natural agents leads to the reversal of epithelial-to-mesenchymal transition in gemcitabine-resistant pancreatic cancer cells Cancer Res 2009 69 6704 6712 10.1158/0008-5472.CAN-09-1298 19654291 PMC2727571

[B25] Zhang J Zhang T Ti X Shi J Wu C Ren X Yin H Curcumin promotes apoptosis in A549/DDP multidrug-resistant human lung adenocarcinoma cells through an miRNA signaling pathway Biochem Biophys Res Commun 2010 399 1 6 10.1016/j.bbrc.2010.07.013 20627087

[B26] Mudduluru G George-William JN Muppala S Asangani IA Kumarswamy R Nelson LD Allgayer H Curcumin regulates miR-21 expression and inhibits invasion and metastasis in colorectal cancer Biosci Rep 2010 31 185 197 20815812 10.1042/BSR20100065

[B27] Saini S Arora S Majid S Shahryari V Chen Y Deng G Yamamura S Ueno K Dahiya R Curcumin modulates microRNA-203-mediated regulation of the Src-Akt axis in bladder cancer Cancer Prev Res (Phila) 2011 4 1698 1709 10.1158/1940-6207.CAPR-11-0267 21836020 PMC3940389

[B28] Bao B Ali S Banerjee S Wang Z Logna F Azmi AS Kong D Ahmad A Li Y Padhye S Sarkar FH Curcumin analogue CDF inhibits pancreatic tumor growth by switching on suppressor microRNAs and attenuating EZH2 expression Cancer Res 2012 72 335 345 10.1158/0008-5472.CAN-11-2182 22108826 PMC3792589

[B29] Liu Z Xie Z Jones W Pavlovicz RE Liu S Yu J Li PK Lin J Fuchs JR Marcucci G Li C Chan KK Curcumin is a potent DNA hypomethylation agent Bioorg Med Chem Lett 2009 9 706 709 19112019 10.1016/j.bmcl.2008.12.041

[B30] Bora-Tatar G Dayangac-Erden D Demir AS Dalkara S Yelekci K Erdem-Yurter H Molecular modifications on carboxylic acid derivatives as potent histone deacetylase inhibitors: Activity and docking studies Bioorg Med Chem 2009 17 5219 5228 10.1016/j.bmc.2009.05.042 19520580

