# Supplementary material for: Pure curcumin decreases the expression of WT1 by upregulation of miR-15a and miR-16-1 in leukemic cells
Source: J Exp Clin Cancer Res. 2012 Mar 27;31(1):27. doi: 10.1186/1756-9966-31-27 (PMC3325897; doi:10.1186/1756-9966-31-27)

**Supplemental Figure 1.** (A): K562 cells were treated with 5, 10, 20 uM pure curcumin for 48 hours, then the mRNA level of WT1 was detected by qRT-PCR. ABL and GAPDH served as different housekeeping for normalization. (B) Primary leukemic cells of 12 AML patients were separated by Ficoll and were treated with 20 uM pure curcumin for 48 hours, then the mRNA levels of WT1 were detected by qRT-PCR. (C) The protein level of WT1 was detected by Western blotting after negative control(N.C), miR-15a and miR-16-1 mimics were transfected into K562 for 48 hours.


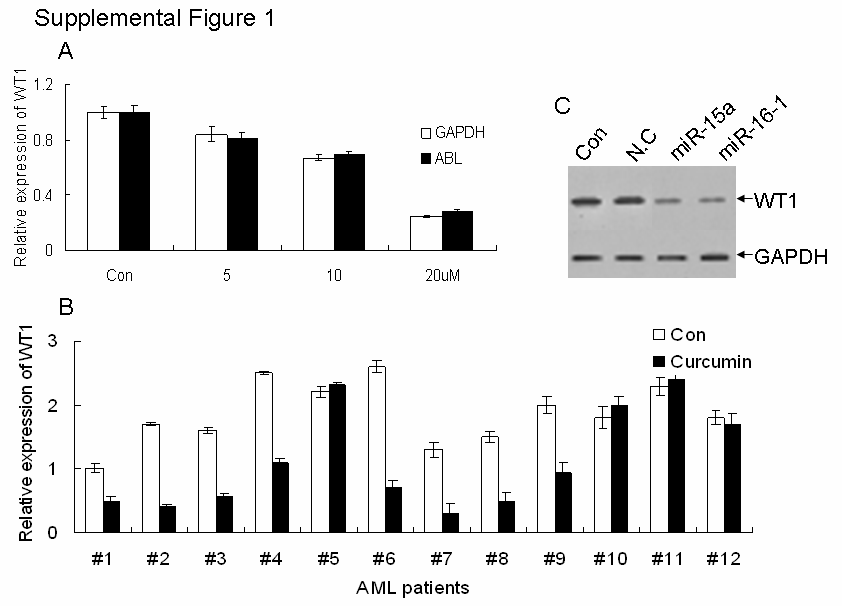


**Supplemental Figure 2.** An illustration of the potential mechanisms of curcumin action in leukemic cells. Curcumin upregulated the expression of miR-15a/16-1 in leukemic cells. Overexpression of miR-15a/16-1 obviously reduced the protein level of WT1. However, downregulation of WT1 by siRNA could not increase the expression of miR-15a/16-1. These events showed that curcumin induced-upregulation of miR-15a/16-1 was an event upstream to the downregulation of WT1. Finally anti-miR-15a/16-1 oligonucleotides (AMO) partly reversed the down-regulation of WT1 induced by curcumin in leukemic cells and reversed the inhibition of cell proliferation caused by curcumin in K562 and HL-60 cells.


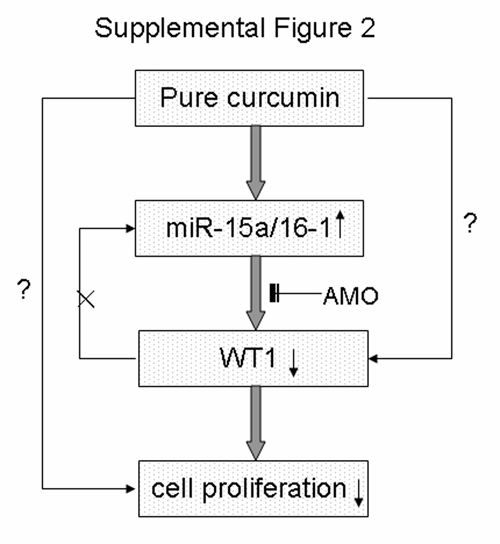

Supplement: Additional file 1 — Figure S1. (A) K562 cells were treated with 5, 10, 20 uM pure curcumin for 48 hours, then the mRNA level of WT1 was detected by qRT-PCR. ABL and GAPDH served as different housekeeping for normalization. (B) Primary leukemic cells of 12 AML patients were separated by Ficoll and were treated with 20 uM pure curcumin for 48 hours, then the mRNA levels of WT1 were detected by qRT-PCR. (C) The protein level of WT1 was detected by Western blotting after negative control(N.C), miR-15a and miR-16-1 mimics were transfected into K562 for 48 hours. Figure S2. An illustration of the potential mechanisms of curcumin action in leukemic cells. Curcumin upregulated the expression of miR-15a/16-1 in leukemic cells. Overexpression of miR-15a/16-1 obviously reduced the protein level of WT1. However, downregulation of WT1 by siRNA could not increase the expression of miR-15a/16-1. These events showed that curcumin induced-upregulation of miR-15a/16-1 was an event upstream to the downregulation of WT1. Finally anti-miR-15a/16-1 oligonucleotides (AMO) partly reversed the down-regulation of WT1 induced by curcumin in leukemic cells and reversed the inhibition of cell proliferation caused by curcumin in K562 and HL-60 cells. [file 1756-9966-31-27-S1.DOC]
